# Research on the Impact of the Built Environment on the Characteristics of Metropolis Rail Transit School Commuting—Take Wuhan as an Example

**DOI:** 10.3390/ijerph18189885

**Published:** 2021-09-20

**Authors:** Jiandong Peng, Jiajie Qi, Changwei Cui, Jinming Yan, Qi Dai, Hong Yang

**Affiliations:** 1 School of Urban Design, Wuhan University, Wuhan 430072, China; 00006709@whu.edu.cn (J.P.); 2014301530106@whu.edu.cn (J.Q.); 2014301530087@whu.edu.cn (C.C.); 2 Guangzhou Planning and Design Survey Research Institute, Guangzhou 510030, China; moveingto@gmail.com; 3 Wuhan Transportation Development Strategy Institute, Wuhan 430014, China; sty_hust@163.com

**Keywords:** rail transit, built environment, travel behavior, school commuting, Wuhan

## Abstract

The long-distance commute to school caused by urban sprawl and the car-oriented urban construction model are key factors leading to primary/middle school students being picked up by their parents in cars. Encouraging those students to take rail transit can reduce their dependence on cars. This paper uses a stepwise regression based on rail-transit swipe data to explore the influence of the built environment on rail-transit commuting characteristics in Wuhan, and uses a geographically weighted regression (GWR) model to analyze the spatial heterogeneity of significant influencing variables. The study found that: (1) 60% of students are one-way commuters; (2) 88.6% of students travel less than 10 km; (3) the floor area ratio, bus station density and whether the station is a transfer station have an obvious positive effect on the flow of commuters; (4) whether the station is a departure station has a positive effect on the commuting distance, but the mixed degree of land use and road density have a negative effect on the commuting distance. This research can assist cities in formulating built environment optimization measures and related policies to improve school-age children’s use of rail transit. This is important in the development of child-friendly cities.

## 1. Introduction

School commuting refers to the behavior of urban elementary and middle school students who go to school and go home from school, with two forms: children going to school and going home alone and children who are picked up and dropped off by others. For a long time, countries around the world have been committed to creating “child-friendly cities” and encouraging primary and middle school students to adopt the active school mode of walking and cycling. This not only helps reduce the use of urban cars, but also benefits the physical and mental health of elementary and middle school students [1,2]. However, with the expansion of urban space and the uneven distribution of educational resources, some elementary and middle school students travel too far, which is also an important factor that causes parents to use cars to pick up their students. Therefore, encouraging middle- or long-distance students to use rail transit to travel alleviates urban traffic and improves safety around elementary and middle schools [3].

A large number of studies have shown that residents’ travel behavior is affected by the built environment [4,5,6]. In contrast, children’s resistance to external risks is low, which makes children’s school-commuting behaviors more susceptible to the impact of the built environment. Existing studies have found that the distance to school, street connectivity and safety, and school location have an important influence on whether children will choose to travel [7]. The closer the distance to school, the higher the proportion of children who actively go to school. Although the “Residential District Code Design GB 50180-2018” stipulates a service radius of 500 m for elementary school and 1000 m for middle schools, due to China’s school district policy, some families will choose to buy school district houses with poorer construction quality to obtain better education resources and live further away. When the school distance exceeds 1.3 km, the chance of children actively going to school significantly decreases [8]. The higher the safety of the street, such as their having surveillance and sidewalks, the more conducive they are to children actively going to school [9]. However, the correlation between street connectivity and children’s active school commuting behaviors is inconsistent in different countries. Some scholars believe that the higher the street connectivity, the better the streets are for walking [10,11], Others believe that higher street connectivity is likely to increase motor vehicle use, and thus affect the safety of streets. The higher the degree of street connectivity, the more likely it is that there is increased use of motor vehicles, affecting the safety of the street [12,13,14]. This is due to the different traffic policies of different countries. Compared with the suburbs, the proportion of students who commute to central urban area schools actively is higher than that in the suburbs. This may be due to the closer distance to schools in the central area and the more mature built environment around the school [8,15]. In addition, the mixed degree of land use, residential density, and personal characteristics such as children’s age and gender have an impact on whether children actively travel [16,17].

The main modes of transportation that children use to actively get to school include: walking, bicycling, and bus and rail transit. Most of the existing research was conducted from the perspective of children walking or riding a bicycle, and the research on children’s use of rail transit to commute to school is insufficient. Conducting research related to school commuting behavior and rail transportation complements the existing research. Cities in China have safer urban environments, lower crime rates and high-density urban rail networks, promoting the use of rail transit for school-age children. Studies have shown that the convenience of rail transit may increase the probability of children actively travelling. The density of the station distributed near the school and community and the distance to the station also affect whether children and their families use rail transit for commuting [18,19]. Therefore, exploring the impact of the built environment on rail-transit school commuting is very important for improving children’s active commuting rates and improving the use of rail transit. In traditional research, schools in different regions are usually selected and questionnaire surveys offered to students. Although the questionnaire survey can accurately reveal the interviewee’s school commute and obtain other information (such as personal socioeconomic characteristics), it also has shortcomings, such as small samples, high cost, and unstable accuracy. More importantly, it is often difficult for children to accurately describe and evaluate their perception of the built environment and its impact on their school commuting behavior. With the application and development of big data and national preferential policies for students to take rail transit, in studies related to children’s commuting behavior, some researchers have used credit card data to identify and analyze the characteristics of elementary and middle school students’ rail-transit commuting behavior [3], but there is a lack of research on the differences in children’s choice of the subway when commuting to school [20].

In local contexts, cities in China, especially those with high-density public transportation-oriented development such as Wuhan, are significantly different from cities in Europe. These cities have higher population density and more severe surface traffic congestion due to high-density development, but the cities provide better quality and safer public transportation services that facilitate children’s use of public transportation. Wuhan’s urban pattern of “two rivers intersecting and three towns standing” has formed a unique build environment and school commuting characteristics. Due to the natural landscape, the density of the road network varies greatly from one area to another. The main cross-river corridors are obviously congested and the road traffic pressure is high. Rail transit is gradually becoming an effective way to relieve traffic congestion and solve the problem of medium- and long-distance school commuting due to its characteristics of high speed, high capacity, frequency, punctuality, safety and comfort. As of 2019, nine rail lines have been opened in Wuhan, with a total line network of 313 km. The rail-transit line network accounts for more than 40% of the proportion of total public transport passenger traffic and the sharing rate of cross-river passenger flow in Wuhan. Rail-transit stations cover more elementary school and middle schools, basically covering all key schools in Wuhan. In a survey of Chinese scholars [21], it was found that more than 90% of parents and 60% of children perceived the area around rail stations to be safer, and although there were some differences in environmental perceptions between children and parents, most families perceived rail transit to be safer. For these reasons, the choice of rail transportation for commuting to school is higher.

Based on this, this paper explores the characteristics of metropolis rail-transit school commuting based on rail-transit credit-card data in the central area of Wuhan. A stepwise regression analysis is used to explore the influence of the built environment and the station factors that affect the characteristics of urban rail-transit students, and a GWR model is used to analyze the spatial heterogeneity of these influences in more depth, so as to more scientifically and effectively assist in urban planning and land-use policy formulation at the microscopic scale, and to provide a reference for the construction of a “child-friendly society”.

### 1.1. Factors Influencing the Behavior of School Commuting

The difference between children’s school commuting patterns and adult commuting and business travel is that children’s travel patterns are more influenced by family decisions and are correlated with family members’ activities [22,23]. Family members are more likely to transport children when they have a flexible work time or no work [7]. Children are also more likely to be transported if their commuting paths to school coincide with their parents’ commuting paths, and priority is given to the more convenient path to transport the children. In addition, the economic status of families also has an impact on children’s travel patterns. The better-off families tend to use motor vehicles to travel, and new bus lines, the planning and construction of kindergartens near rail-transit stations and adjusting the layout of kindergartens and elementary and middle schools are conducive to children and families using public transportation such as rail transit to travel [24].

In addition, the built environment affects children more than adults. Some scholars have evaluated the impact of the built environment on school travel, looking at three aspects: neighborhood environment, school and surrounding built environment, and school-path built environment. On this basis, many scholars have used the social–ecological modeling theory and methods to analyze school commuting behavior and found that proximity, safety, connectivity, comfort and attractiveness, and social capital all have an impact on children’s school commuting choice [25,26].

At the policy level, many countries have taken steps to optimize infrastructure to achieve ease and equity in school commuting. In China, the nearby enrollment policy is the biggest policy that affects school commuting. However, the policy has not been fully implemented since its release, mainly due to the uneven distribution of educational resources and the resulting and increasingly serious problem of school district housing [27]. A school district house is a house near a key school, and families that own these properties can enter a key elementary and middle school without a test [28]. In general, the families of students who attend key schools are generally better off than those who attend average schools, with a higher proportion of families owning cars and using motorized transportation. While children are not mature enough to make their own travel decisions due to their age and mental maturity, their school commuting method is often decided by their parents [29]. This leads to a greater proportion of students in key schools commuting to school by motor vehicle, which, in turn, leads to traffic congestion problems and parking difficulties that can lead to complex traffic conditions near schools and have a great impact on the safety of students [30]. However, at the same time, some of the school district houses are old, so families who buy school district houses may live in other places [31], resulting in students crossing administrative districts over long distances to commute to school.

### 1.2. The Influence of the Built Environment on School Commuting Behavior

The built environment refers to a variety of buildings and places that have been artificially constructed and modified, as opposed to the natural environment, and usually contains three components: land use, transportation systems, and urban design [32]. Compared to the commuting behavior of adults, the commuting behavior of children can be focused on the spatial scale of the residence, the school and the paths between them [33], which impact five main areas: density, diversity, design, accessibility of facilities and distance to the center [32,34].

Density has a more significant effect on school commuting than the diversity and design factors [35]. Current research shows that high residential density significantly affects school commuting behavior [36]. The higher the residential density, the greater the number of schools, and the shorter the potential distance to school, facilitating children’s use of slow-moving systems or public transportation for related activities [37].

The higher the mixed degree of land use, the more balanced the “residential-educational” situation in the area, the higher the density of land-use types related to living and leisure, and the higher the possibility of students walking to school [38,39]. On the contrary, areas with a lower mixed degree of land use tend to have an imbalance in the “residential-educational” situation in the area, with relatively long distances to travel to school, and parents tend to use more efficient and safer motorized transportation. In China, areas with a higher mixed degree of land use tend to be located in central urban areas, which have advantages over suburban areas in terms of their built environment and distance to school, and students are more likely to commute by walking and using public transportation.

The micro-level design of street connectivity, neighborhood scale, and road network structure in urban design can also have an impact on children’s school commuting behavior. As an important way of encouraging children’s walking, sidewalks have an important impact on children’s adoption of walking, as does their pavement integrity, continuity, width, etc. [40]. As well as convenient sidewalks, a walkable neighborhood scale and square grid road network layout also can create a safe, comfortable, and convenient green travel environment, thus increasing the possibility of children using non-motorized and public transportation to commute to school [41]. It has also been shown that the connectivity of commuting routes with road density and intersection density also has a relevant effect on children’s adoption of non-motorized transportation trips [12,41]. In terms of the accessibility of facilities, distance to public transportation stations and the ease of public transportation connections and transfers had a significant effect on children’s use of public transportation to commute to school. The lack of alternative modes of transportation in the vicinity of the residence and school results in children’s parents using private cars for transportation. The more often the public transportation stops within a reasonable walking distance, the higher the convenience of public transportation connections and transfers, and the higher the diversity of transportation modes, the higher the proportion of children that use public transportation for travel [42].

Distance to the central area is an indicator of regional accessibility. Residential location and school location have an impact on how children travel to school. Children living in urban centers are more likely to use public transportation, such as rail transit, to get to school than children living in suburban areas, but as the distance from the center increases, children are more likely to use rail to get to and from school [3].

## 2. Materials and Methods

### 2.1. Study Area and Data Sources

#### 2.1.1. Study Area

**Figure 1:**
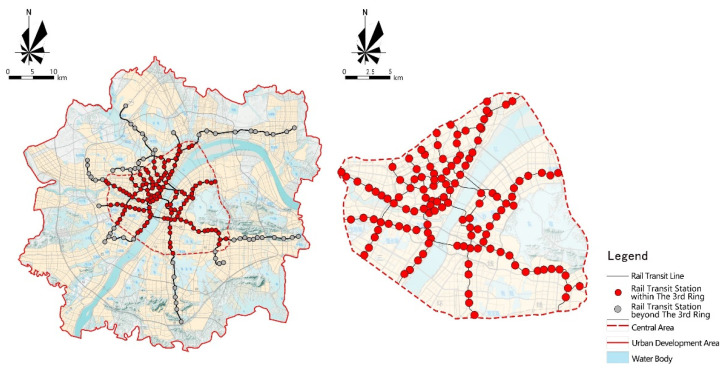
2019 Wuhan City rail transit stations and line distribution.

The research object is Wuhan, the largest city in central China and the famous education capital of China. The study area covers the central urban area within the third ring road of Wuhan, including Hanyang, Qiaokou, Jianghan, Jiangan, Qingshan, Wuchang and Hongshan, with a total area of 525 km^2^ and a total population of 9,236,300 in 2018, accounting for 83.35% of the resident population of Wuhan. There are 276 elementary school, 187 middle schools and about 520,700 students in the region, with a dense distribution of elementary and middle schools and frequent school commuting behavior, which is highly typical. The scope of the study area is shown in Figure 1.

#### 2.1.2. Scope Definition of the Area around the Rail Transit Station

Referring to previous studies, the area around the 800 m radius of a rail-transit station is usually set as the maximum range of station area influence for that station. However, because students’ physical functions are still immature compared with adults, and they have some differences to adults in terms of stride length and speed, the area around the rail-transit station area for students needs to be defined [43,44]. According to the travel survey, the walking speed of students is about 90% of that of adults, i.e., 1.1 m/s. Therefore, this paper sets the area of 720 m radius around the rail-transit station as the area of influence of the station.

#### 2.1.3. Data Sources and Pre-Processing

The data used in this paper include: (1) urban rail-transit credit-card data for one consecutive week in March 2019, including station name, latitude and longitude coordinates, station type, and number of entrances and exits (as shown in Table 1); (2) Wuhan house price data in March 2019, including residential type, address, greening rate, plot ratio, property fee, and corresponding school district information; (3) Wuhan land-use status data in 2015; (4) Wuhan POI (Point of Interest) data in 2019, including spatial location information of various facilities in Wuhan such as primary and secondary schools; (5) Wuhan community population distribution data in 2018.

This study uses Auto Navi Map Application Programming Interface (AMAP API) to extract the built environment data of the area around rail transit stations, including: station-feature data (distance between stations and Wuhan main and secondary centers), density-feature data (floor area ratio, population density), design-feature data (road network density, road-intersection density), diversity-feature data (mixed degree of land use, density of various facilities), and other types of data, such as house prices.

### 2.2. Research Methods

#### 2.2.1. Technology Route

**Figure 2:**
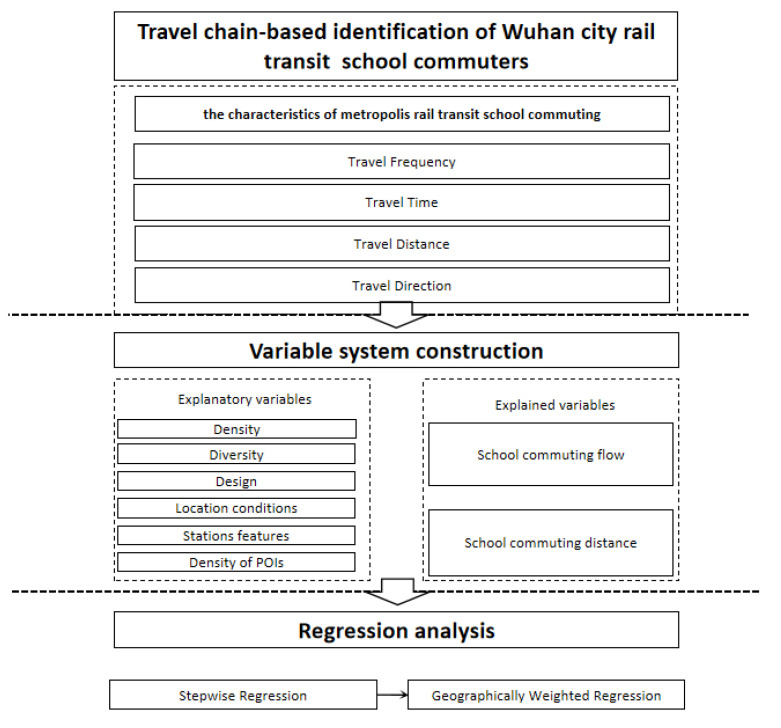
Technology route. POIs: Point of Interests.

This paper firstly identifies rail commuters based on Wuhan rail-transit swipe card data and analyzes the rail-transit school-commuting behavior and spatial pattern. Secondly, the school-commuting flow and the school-commuting distance in the characteristics of metropolis rail-transit school-commuting are taken as the explanatory variables, and the variable system is constructed based on the population, land-use and POI data. The correlation between them is analyzed using stepwise regression and geographically weighted regression, and the technical route is shown in Figure 2.

#### 2.2.2. Variable Settings

With reference to previous studies, this paper uses school-commuting flow and distance as dependent variables to represent the school-commuting characteristics of urban rail transit. Among them, the school-commuting flow is expressed as the number of students at each station, and the school-commuting distance is expressed as the average school-commuting distance of students at each station. In order to examine the influence of the built environment on the commuting characteristics of urban rail transit, 10 independent variables involving the built environment, urban rail-transit station characteristics and school and residential location were selected for analysis in this paper (Table 2).

#### 2.2.3. Model Selection

- Stepwise Regression Mode

The stepwise regression establishes the “optimal” multiple linear regression equation by introducing the significant independent variables one by one, testing the old independent variables one by one after the introduction of the new variables, eliminating the insignificant variables, and repeating this step until no new variables are introduced and no old variables are eliminated. The formula is as follows:

(1) $$y=\beta_{0}+\beta_{i}x_{i}+\varepsilon \;\;\;i=1,2,\ldots ,n$$

Calculate the test statistic F of the n regression coefficients and find the maximum value as $F_{i1}^{1}$, if $F_{i1}^{1}\geq F_{\alpha }$, then introduce $x_{i1}$ into the model and construct a binary regression model of y with a subset of independent variables, calculate the value of the test statistic of the regression coefficients F of the variables and choose the maximum value of it as $F_{i2}^{2}$, if $F_{i2}^{2}\geq F_{\alpha }$, then continue to iterate until the maximum value of F of the independent variables is less than the critical value.

- Geographically Weighted Regression Model

Since the regression coefficients of the global model do not present enough spatial differences [45], the GWR model can account for the spatial autocorrelation and spatial heterogeneity of the data by embedding the spatial structure into the regression model, whose regression coefficients are a function of spatial location. The formula is as follows:

(2) $$y_{i}=\beta_{i0}\left(u_{i},\nu_{i}\right)+\overset{p}{\underset{k=1}{\sum }}\beta_{ik}\left(u_{i},v_{i}\right)x_{ik}+\varepsilon_{i}\;\;\;i=1,2,\ldots ,n$$

$\beta_{i0}\left(u_{i},\nu_{i}\right)$ is the estimated intercept of the ith site; P is the number of independent variables; $x_{ik}$ is the kth independent variable of the ith site; $\beta_{ik}\left(u_{i},v_{i}\right)$ is the regression parameter of the independent variable $x_{ik}$; $\varepsilon_{i}$ is the random error of the ith site.

## 3. Results and Discussion

### 3.1. Spatial and Temporal Characteristics of Wuhan’s School Commuting Behavior

#### 3.1.1. School Commuting Patterns

Due to the existence of parental pick-up and drop-off behaviors, students taking the subway are likely to have the situation of only going or only returning [3]. In this paper, the commuting patterns of students taking rail transit are divided into four modes: coming and going, only going but not returning, only returning but not going and other modes, and when one of the commuting modes accounts for more than 60% of the commuting modes identified by the credit card data in a consecutive week, it is recognized as the commuting pattern of the students.

According to the statistics and calculations, 36.3% of the students commute to school in the coming and going pattern, 27.1% in the only returning pattern, 32.8% in the only going pattern, and 3.7% in the other mode. Nearly 60% of students commute to school for one way by rail transit, and the situation of parents picking up and dropping off their children is more common in Wuhan, which is in line with the actual situation at this stage.

#### 3.1.2. Travel Frequency

**Figure 3:**
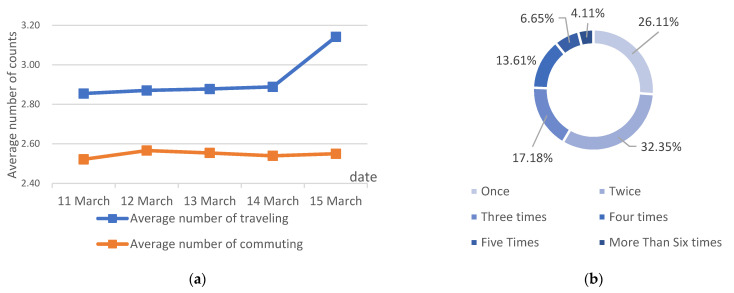
Traveling and commuting frequency. (**a**) Frequency of students traveling and commuting on school days; (**b**) Average number of school commuting as a percentage.

The average number of rail-transit traveling behaviors per student on a school day is 2.93 per day, with an average number of rail-transit commuting behaviors of 2.55 per day. The average number of school-commuting behaviors on a school day is relatively stable, with the average number of traveling behaviors peaking on Fridays, probably due to a significant increase in the number of students doing other activities such as recreation, on Friday evenings. For the school-commuting frequency, 58% of students have an average of two or fewer daily rail-transit commuting behaviors, with 11% of students having an average of four or more rail-transit daily commuting behaviors. Traveling and commuting frequency are shown in Figure 3.

#### 3.1.3. Travel Time

**Figure 4:**
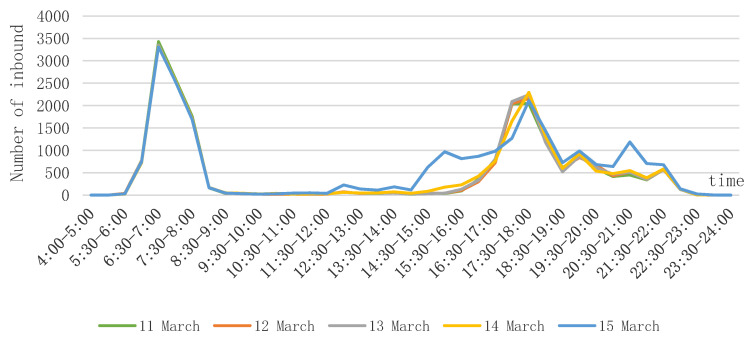
Distribution of departure time of school commuting.

The departure time is influenced by the school time and after-school time of elementary and middle schools, which is highly concentrated. The morning peak of school students in Wuhan who take rail transit to school appears at 6:00–8:30, the evening peak appears at 16:00–18:30, and the noon peak is not obvious. Compared with the evening peak, the morning peak has a higher concentration, which is due to the fact that the school times are basically the same in the morning, but there are differences in the after-school times in the evening, and there are two small peaks in the evening from 19:00–19:30 and 21:30–22:00, which are caused by some of the senior students’ evening self-study. The reason for the insignificant noon peak is that most of the students who take commute to school by rail transit have a long distance between their schools and their places of residence, and they have a tight lunchtime, so they rest at school. Observing the five-day distribution, the school-commuting departure moments from Monday to Thursday show similar characteristics, but the Friday evening peak is earlier and more volatile, caused by the early after-school time of some schools, such as boarding schools, and a new peak hour at 20:30–21:00, due to the increase in students conducting leisure and recreational activities on Friday evening. The distribution of departure time of school commuting is shown in Figure 4.

**Figure 5:**
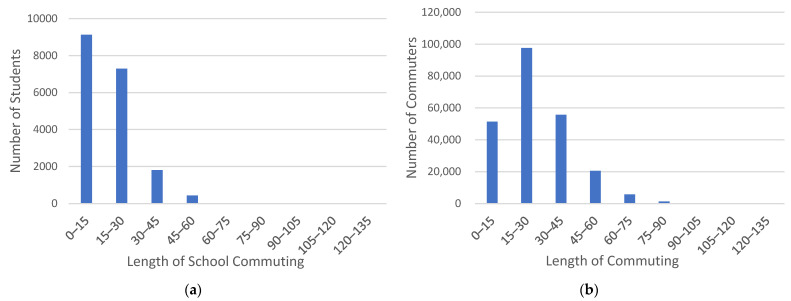
Distribution of students’ and commuters’ length of commuting. (**a**) Distribution of students’ length of commuting; (**b**) Distribution of commuters’ length of commuting.

The length of commute reflects the distance between students’ residence and school. Compared with commuters, students’ length of commute is concentrated within 15 min, accounting for 48.9% of students, and students with a commute length of 30 min account for 88.0% of the total. The largest percentage of students, 24.1%, have a length of 11–15 min, or 4–5 stops from their place of residence to school, and 1.14% still have a length of 50 min or more. The distribution of students’ and commuters’ length of commuting are shown in Figure 5.

#### 3.1.4. Travel Distance

**Figure 6:**
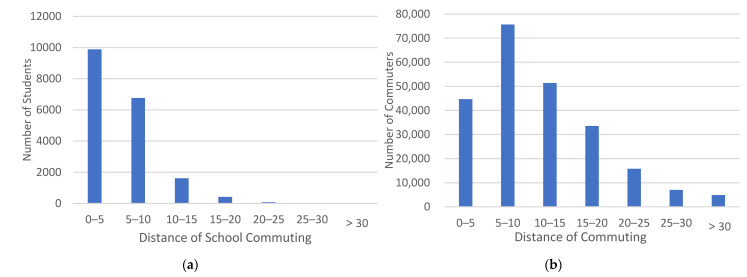
Distribution of students’ and commuters’ distance of commuting. (**a**) Distribution of students’ distance of commuting; (**b**) Distribution of commuters’ distance of commuting.

The daily route choice and range of activities of students who commute by rail transit on school days are relatively fixed, with 52.6% of students commuting within 5 km and 88.6% within 10 km. Compared to the distance traveled by commuters, nearly 90% of students mainly travel short to medium distances, but 11.4% of students still need to take rail transit to go to school for more than 10 km daily. The distribution of students’ and commuters’ distance of commuting are shown in Figure 6.

#### 3.1.5. Travel Direction

**Figure 7:**
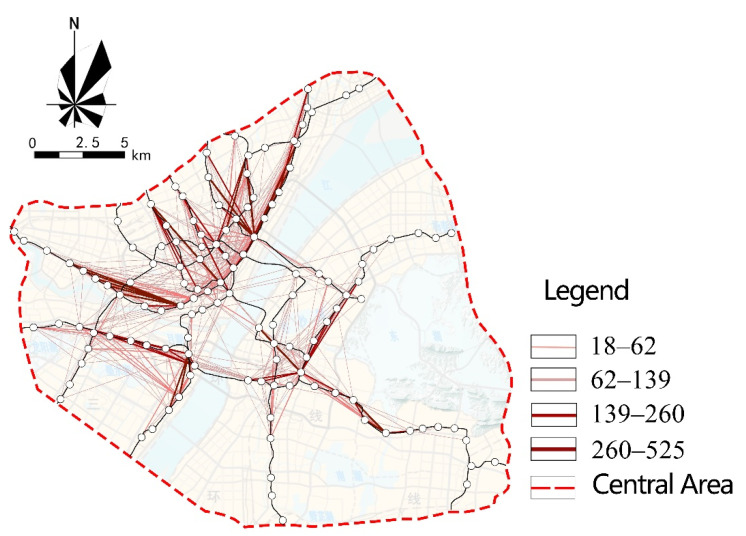
Distribution of rail transit school commuting direction.

**Figure 8:**
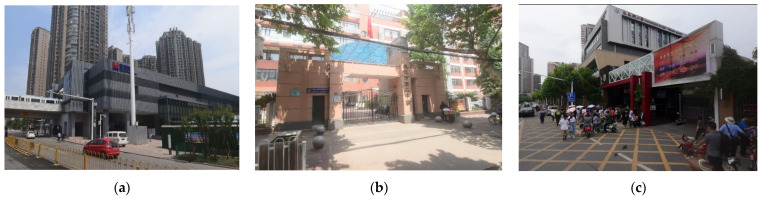
Huangpu area build environment photos. (**a**)Huangpu Road rail transit station; (**b**) Wuhan No.2 High School; (**c**) Changchun Street Primary School.

In terms of direction, the school commuting behavior is more concentrated, and dominated by short and medium distances. Figure 7 shows the distribution of rail transit school commuting direction. As shown in Figure 7, there are five concentrated areas of through-schooling, as shown in Table 3. These areas are located in the central area of Wuhan, with obvious location advantages and a superior built environment, which generates a higher school commuting flow compared with other areas. Additionally, more importantly, there are more key elementary schools and middle schools distributed in the area of Wuhan, which not only generate more frequent school-commuting links with the surrounding stations, but also generate long-distance school-commuting behavior. Figure 8 shows the built environment of Huangpu Road as a typical area.

### 3.2. The Influence of Built Environment Factors on the School Commuting Flow

#### 3.2.1. Analysis of Factors Influencing the Built Environment on School Commuting Flow

Table 4 shows that the floor area ratio, bus station density and whether the station is a transfer station have an obvious effect on the flow of commuting to school. Transfer stations tend to have higher regional accessibility compared to non-transfer stations, so the stations have a higher school commuting flow. In addition, rail transit stations in the central city of Wuhan are mainly located on both sides of the Yangtze River and The River Han, an area where elementary and middle schools are partially concentrated. Taking Huangpu Road transfer station as an example, there are 10 elementary and middle schools around the station, so its rail-transit school-commuting flow is much higher than the surrounding non-transfer stations. The higher the floor area ratio, the higher the number of potential school-age children, which has an impact on the flow of school commuting. At the same time, the floor area ratio of Wuhan shows a general decreasing trend along the central urban area to the suburbs, and the central urban area has advantages in terms of diversity and safety, which is more conducive to students taking rail transportation to school. Additionally, the traffic congestion problem in this area is more serious than in other areas, which also leads students to take non-motorized transportation to school. The density of bus stops increases the convenience of students using other means of transportation. The higher the density of bus stops, the higher the flow of school commuting, which is also consistent with the conclusions of existing related studies [18,19].

In addition, a stepwise regression of the evening peak and morning peak in school-commuting flow shows that parking lot density has a significant effect on the evening peak through-school flow. The density of the parking lot also has a significant impact on the flow of school commuting in the evening peak. If there is no parking lot near the school, the probability of parents picking up their children will be greatly reduced.

#### 3.2.2. Analysis of the Spatial Heterogeneity of Factors That Have a Significant Effect on School Commuting Flow

**Figure 9:**
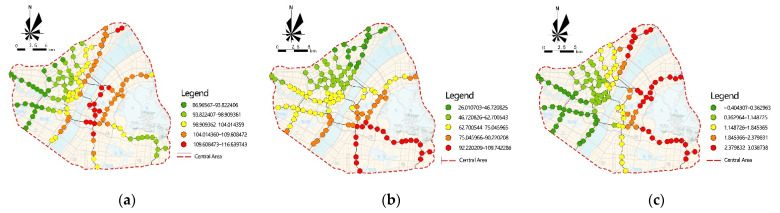
The spatial variation in the impact of significant variables on school commuting flow. (**a**) whether station is a transfer station regression coefficient; (**b**) floor area ratio regression coefficient; (**c**) bus stop density regression coefficient.

Figure 9 shows the spatial variation in the impact of significant variables on school commuting flow.

The existence of a transfer station has a significant positive influence on rail-transit school-commuting flow, but its influence effect shows differences in space. The spatial distribution of rail-transit transfer stations and the distribution of elementary and middle schools in Wuhan generally show a trend of high density in Hankou and low density in Wuchang and Hanyang. The transfer stations in Hankou can cover most of the schools, so the transfer stations’ flow of school commuting in Hankou is much higher than the transfer stations in other areas. Wuchang is restricted by topography, and the coverage rate of schools by rail transit is relatively low, so the transfer stations’ flow of school commuting is relatively small. Hanyang is lagging in termso f rail-transit construction, and the total population and the number of schools in this area are the lowest, so the transfer stations’ flow of school commuting is the smallest.

The floor area ratio of Wuchang remains in the mid-range in Wuhan, but the school commuting flow is greatly affected by it. This is due to the early construction of Wuchang and the high proportion of residential buildings in the area. Although the residential building floors are low, the building density is high, and thus many students live here. Due to the lack of high-quality educational resources in the area, some students have to study in Hankou, and are subject to the Yangtze River and other natural geographical barriers. This means that rail transit is the best choice for students in the area to commute to school. While the floor area ratio of Hankou is higher, the mixed degree of land use around the rail-transit stations is higher, so there are more people who live there for business and the number of students is not significantly higher than Wuchang. At the same time, the distribution density of key schools in this area is higher, and the density of the regional road network is also higher, so the ways to school are more diversified, and the probability of walking to school or using other means of transportation is greatly increased.

Wuchang’s rail-transit stations’ school-commuting flow is more affected by bus stop density. Due to the natural conditions of the lake and other barriers in this area, the rail-transit network is imperfect and the coverage rate is low. The students living in the surrounding areas must be able to reach the nearby rail-transit station or commute by bus in order to reach the key schools in Hankou or other places with a certain distance. The interchange between bus and rail transit is obvious, so the higher the density of bus stops, the greater the school-commuting flow. Similarly, the areas near the end of the Line 1 and Line 21 are located in the urban fringe, where the rail-transit coverage rate is low, producing the same situation. In contrast, Hankou and Hanyang have a larger rail-transit network, and high coverage of stations near residential areas, which provides more selectivity. Additionally, the interchange between bus and rail transit is weaker, so the impact of the density of bus stops in the region on the school-commuting flow of rail-transit stations is smaller compared to the eastern region.

### 3.3. The Influence of Built Environment Factors on the School Commuting Distance

#### 3.3.1. Analysis of Factors Influencing the Built Environment on School Commuting Distance

Table 5 shows that whether a station is a departure station, road density, and the mixed degrees of land-use have a significant effect on the school-commuting distance using rail transit. Since rail transit departure stations are generally located at the edge of the city and have a longer geographical distance to the central area, students who commute from there have a longer distance to school than other students. The areas with a higher mixed degree of land use and higher road density are concentrated in the central area of Wuhan, and students in these areas can commute over shorter distances to more comfortable and safer schools than those near the third ring road. Students living in suburban areas, which have a lower mixed degree of land use and lower road density, have longer distances to commute to schools than those with a higher mixed degree of land use in the surrounding area.

#### 3.3.2. Analysis of the Spatial Heterogeneity of Factors That Have a Significant Effect on School Commuting Distance

**Figure 10:**
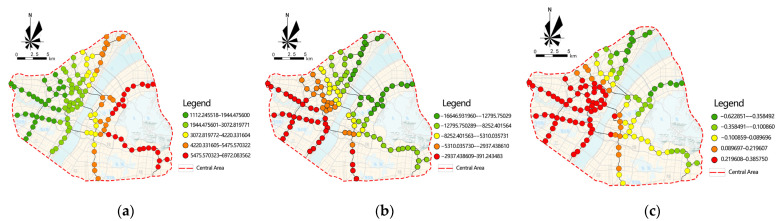
The spatial variation in the impact of significant variables on school-commuting distance. (**a**) whether station is a departure station regression coefficient; (**b**) mixed degree of land-use regression coefficient; (**c**) Road-density regression coefficient.

Figure 10 shows the spatial variation in the impact of significant variables on school-commuting distance.

Whether a station is a departure station has a significant positive influence on the school commuting distance, but it shows a strong heterogeneity in space. Among them, the rail transit located in Wuchang is radially distributed, and the closer the departure station is, the farther it is from the central area, which has a longer geographical distance compared to other stations. The advantage of rail transit is efficient and stable, making it the first choice for long-distance commuting. Similarly, Line 21 and Line 1 have a relatively large impact. In contrast, Hankou and Hanyang’s rail transit is distributed in a network pattern, so the average distance of each station from the central area is shorter; thus, whether it is a departure station has a relatively low impact on the distance to school.

Due to the time of construction, the government and the public recognize the importance of the balance between work and residence, so the development of suburban new towns, which are relatively late in construction, is oriented toward the balance of work and residence, meaning that the mixed degree of land use is higher. However, because the built environment of these stations is much lower than that of the central area in terms of comfort and safety, the probability of students choosing to study here is also lower, and commuting behavior still occurs between the residential area and the high-quality schools in the central area. Therefore, the distance to school in Wuchang, Line 21 and the area at the end of Line 1 are more influenced by the mixed degree of land use. While the mixed degree of land use of Hankou and Hanyang, which are relatively close to the central area, is also higher, they have a closer geographical distance, and the distance to schools is significantly shorter, so the influence of the mixed degree of rail-transit stations’ land use on the distance to school is smaller in this region.

Wuchang, Line 21 and the area at the end of Line 1 are farther away from the central area, the surrounding areas are late in construction, and although the regular road network is planned, the road density is still significantly lower than that in Hankou. At the same time, due to the lack of quality educational resources, increasing the density of the road network in one small area alone has a relatively small moderating effect on the behavior of commuting to school in the central area, with a medium or long distance. In contrast, Hankou has a higher road network density and higher road connectivity, which means that stations are more accessible, especially along the river. This leads to a more typical spatial organization of high road-network density and small-scale neighborhoods. Compared with Wuchang, the areas with lower road density in Hankou are closer to the central area, so the distance to school is shorter.

## 4. Conclusions

Taking the central area of Wuhan as an example, this paper explores the influence of the built environment on the characteristics of rail-transit school commuting based on multi-source big data such as urban rail-transit credit-card data, house-price transaction data and current land-use data, using descriptive statistical analysis, stepwise regression model and GWR model. From the results of the above analysis, the following conclusions can be drawn.

Based on the analysis of rail-transit credit-card data, it was found that there are four types of school-commuting patterns for students who take rail transit to school, of which nearly 60% are one-way trips, and the situation of parents picking up and dropping off their children to and from school is common. Similar to commuters, students who commute to school by rail transit have a high degree of regularity, with an early morning peak and an evening peak. The evening peak lasts longer compared to the morning peak due to the different school and after-school times. In terms of travel distance and travel time, nearly 90% of the students commute within 30 min, mainly for short and medium distances, and the routes are relatively fixed, but there is still a very small number of students who commute for more than one hour. In the direction of school commuting, five typical commuting areas were formed, namely Huangpu, Zhongjiacun, Chongren, Zhongnan and Changgang Road, with a dense distribution of key schools and frequent commuting behaviors in the area.

Based on the stepwise regression analysis, it was found that there are differences in the factors influencing the built environment, as well as the school-commuting flow and distance. Among them, whether the station is a transfer station, bus stop density, and floor area ratio have significant effects on rail-transit school-commuting flow, and whether the station is a departure station, road density, and mixed degree of land use have significant effects on rail-transit school-commuting distance. It was found that bus stop density influenced the school-commuting flow by affecting the transfer behavior, which is consistent with the findings of other scholars. In addition, by conducting stepwise regressions on the morning and evening peak flows, respectively, it was found that parking lot density has a significant effect on school-commuting behavior, and parking lot density could significantly affect the frequency of parents picking up and dropping off their children at school. By adjusting the number of parking lots around schools, the occurrence of school commuting by private cars could be reduced to a certain extent.

Based on the GWR model analysis, there are significant spatial differences in the effects of the built environment on rail-transit school-commuting flow and distance. Whether the station is a transfer station, floor area ratio, and bus stop density have a significant positive effect on the school-commuting flow. Compared with Wuchang, Hankou has more high-quality teaching resources around the transfer stations, and thus has a higher school-commuting flow; students in the high-floor-area ratio of Hankou have more diverse means of commuting to school, and the proportion of students taking rail transit at the same level is relatively low. Additionally, the transfer effect of bus stops is more obvious, due to the radial network distribution in Wuchang, so the school-commuting flow in Wuchang is more affected by the density of bus stops. The mixed degree of land use and road density have a significant negative effect on the distance to school, while whether the station is a departure station has a positive effect. The departure stations in Hankou and Hanyang are closer to the central area, and thus have shorter school-commuting distances, and road density shows spatial differences by affecting station accessibility. The new districts, which are farther away, have a higher mixed degree of land use; however, the choices are still high-quality educational resources in the central area, so they are more affected by the mixed degree of land use.

The transportation-oriented development (TOD) mode is the direction in which scholars are now focusing. As an important part of urban public transportation, rail transit plays an important role in solving the problem of long-distance travel and relieving urban traffic congestion. Public-transportation-oriented construction not only solves the problems of residence and employment, it should also meet the usage needs of different people of all ages and bring practical happiness to all kinds of people. Children, as the main beneficiaries of a child-friendly society, are also in need of attention. This study provides data to support a safer, more comfortable, and more pleasant environment for children, and provides directions for optimizing the built environment. As a rapidly developing mega-city, Wuhan city has accelerated the speed of urban road construction, motor vehicle ownership has gradually increased, the pressure on the road network has gradually increased, and traffic accidents are frequent. Student groups, especially younger children, who have a poor sense of self-protection and weak perception of the surrounding environment, need key protection objectives. By adjusting the built environment around the stations and increasing the probability of children taking rail transportation, the proportion of children using road transportation can be significantly reduced, thus reducing injury due to traffic accidents. This is important for improving family well-being and maintaining safe urban operation. This study also contributes to the normalization of epidemic prevention and control after a pandemic. Wuhan, as the largest city in Central China, has a large population flow, and more stringent requirements for epidemic prevention and control. By changing the build environment around the stations, students’ reliance on private cars can be reduced, as well as the density of human flow in key prevention and control areas such as schools, this could fundamentally reduce the mobility of the population. At the same time, due to the traceability of rail transit swipe card data, it would be easy to accurately identify the travel location and time of a specific group of people in times of emergency.

Building a “child-friendly society” is not only an important issue in urban construction, but also a key direction for public transportation. Building a public transportation system that meets the needs of all age groups is an important manifestation of social equity. The results of this study reveal the characteristics of school-commuting flow and distance and the factors influencing the built environment, which can help cities to formulate measures and policies to optimize the built environment to enhance the use of rail transit by school-age children. In addition, a family’s socioeconomic characteristics have an important influence on children’s commuting pattern. In the next study, the absence of these data can be compensated by typical regional research to further verify and analyze the mechanism of each influencing factor.

## Figures and Tables

**Table 1:** Example of Wuhan rail transit smart card data.

Card Number	Time	Line	Station	Swipe Type	Card Type
80271112xxxxxxxx	11 March 2019 06:38:39	1	126	28	1112
80271112xxxxxxxx	11 March 2019 06:57:18	1	119	29	1112

Station 126 of Line 1 is Dijiao, Station 119 is Sanyang Road; swipe type 28 for entering the station, 29 for leaving the station; card type 1112 is elementary and middle school students’ card.

**Table 2:** Variables and data description.

Variable Category	Variable Name	Calculation, Assignment and Interpretation of Indicators
Dependent variable	School commuting flow	Number of students at each station.
	School commuting distance	Average distance to school at each station.
Travel behavior	Travel time	Time of travel.
	Travel frequency	Number of trips during the day.
Location conditions	Location of residence area	Within the 1st Ring Road = 1; Between the 1st and 2nd Ring Road = 2;
	Location of school	Between the 2nd and 3rd Ring Road = 2.
Build environment	Population density	Ratio of the number of people in the range to the total area.
	Floor area ratio	Floor area ratio in the range.
	Bus stop density	Ratio of the number of bus stops in the range to the total area.
	Road density	Ratio of the road length in the range to the total area.
	Road intersection density	Ratio of the number of road intersections in the range to the total area.
	Mixed degree of land use	$S=-\sum_{i=1}^{n}pi*log_{10}pi$ S is the entropy value of the mixed degree of land use, *n* is the number of divisions of land use types, and *pi* is the proportion of the land area in category *i*, $\sum_{i=1}^{n}pi=1$.
	Facilities density	Ratio of the number of facilities in the range to the total area.

**Table 3:** Typical school commuting regional distribution.

School Commuting Regional	Place of Residence	Place of Study	Percentage of
Huangpu area	Danshuichi, Houhu Boulevard, Dazhi Road, Erqi Road, Tazihu, Xingye Road, Xudong, Zhaojiatiao, Zhuyeshan, Xinrong, Zhongyi Road	Huangpu Road	12.43%
Zhongjiacun area	Maying Road, Hanyang Railway Station, Shilipu, Yulong Road, Qilimiao, Qianjincun, Jiangang	Zhongjiacun, Lanjiang Road	9.96%
Chongren area	Gutianerlu, Gutiansanlu, Gutiansilu, Hanxiyilu, Zongguan, Taipingyang	Chongren Road	8.61%
Zhongnan area	Jiyuqiao, Fuxing Road, Shouyi Road, Baotong Temple, Chuhehanjie, Dongting, Qingyuzui, Tieji Road	Zhongnan Road	7.90%
Changgang Road area	Changqing Huayuan, Jinyintan, Wangjiadun East, Xunlimen, Zhongshan Park	Changgang Road, Fanhu	5.10%

**Table 4:** Performance of stepwise regression on school commuting flow.

Variables	Unstandardized Coefficients		*p*-Value
	B	Standard Error	
Constant	−4.251	22.338	0.849
Whether it is a transfer station	79.237	25.649	0.002 *
Bus stop density	5.185	2.357	0.030 *
Floor area ratio	40.998	19.631	0.039 *

* significant at the 5% level.

**Table 5:** Performance of stepwise regression on school commuting distance.

Variables	Unstandardized Coefficients		*p*-Value
	B	Standard Error	
Constant	14220.659	1386.504	0.000
Whether station is a departure station	6237.326	1884.667	0.001 **
Road density	−4513.889	2015.087	0.027 *
Mixed degree of land use	−0.350	0.097	0.001 **

** Significant at the 1% level, * significant at the 5% level.
